# Task-shifting and prioritization: a situational analysis examining the role and experiences of community health workers in Malawi

**DOI:** 10.1186/1478-4491-12-24

**Published:** 2014-05-02

**Authors:** Sarah Smith, Amber Deveridge, Joshua Berman, Joel Negin, Nwaka Mwambene, Elizabeth Chingaipe, Lisa M Puchalski Ritchie, Alexandra Martiniuk

**Affiliations:** 1 Dignitas International, Zomba, Malawi; 2 School of Public Health, University of Sydney, Sydney, Australia; 3 District Health Office, Ministry of Health, Zomba, Malawi; 4 Department of Medicine, Division of Emergency Medicine, University of Toronto, Toronto, Canada; 5 University Health Network, Toronto, Canada; 6 Dalla Lana School of Public Health, University of Toronto, Toronto, Canada; 7 George Institute for Global Health, Sydney, Australia; 8 Sunnybrook Health Sciences Research Institute, University of Toronto, Toronto, Canada

**Keywords:** Community health workers, Lay health workers, Task-shifting, Health promotion, Task- prioritization

## Abstract

**Background:**

As low- and middle-income countries face continued shortages of human resources for health and the double burden of infectious and chronic diseases, there is renewed international interest in the potential for community health workers to assume a growing role in strengthening health systems. A growing list of tasks, some of them complex, is being shifted to community health workers’ job descriptions. Health Surveillance Assistants (HSAs) - as the community health worker cadre in Malawi is known - play a vital role in providing essential health services and connecting the community with the formal health care sector. The objective of this study was to understand the performed versus documented roles of the HSAs, to examine how tasks were prioritized, and to understand HSAs’ perspectives on their roles and responsibilities.

**Methods:**

A situational analysis of the HSA cadre and its contribution to the delivery of health services in Zomba district, Malawi was conducted. Focus groups and interviews were conducted with 70 HSAs. Observations of three HSAs performing duties and work diaries from five HSAs were collected. Lastly, six policy-maker and seven HSA supervisor interviews and a document review were used to further understand the cadre’s role and to triangulate collected data.

**Results:**

HSAs performed a variety of tasks in addition to those outlined in the job description resulting in issues of overloading, specialization and competing demands existing in the context of task-shifting and prioritization. Not all HSAs were resistant to the expansion of their role despite role confusion and HSAs feeling they lacked adequate training, remuneration and supervision. HSAs also said that increasing workload was making completing their primary duties challenging. Considerations for policy-makers include the division of roles of HSAs in prevention versus curative care; community versus centre-based activities; and the potential specialization of HSAs.

**Conclusion:**

This study provides insights into HSAs’ perceptions of their work, their expanding role and their willingness to change the scope of their practice. There are clear decision points for policy-makers regarding future direction in policy and planning in order to maximize the cadre’s effectiveness in addressing the country’s health priorities.

## Background

Globally, 57 countries face critical heath workforce shortages and more than four million health workers are needed to fill this gap [1]. Such shortages of human resources for health combined with the double burden of infectious and chronic diseases contribute to increased mortality and morbidity, impede the achievement of the health-related Millennium Development Goals, and hinder economic growth in low- and middle-income countries. Given this human resource gap, particularly in rural areas, there is renewed interest globally in the potential for community health workers (CHWs) to take on an expanded role in strengthening health systems [1].

Many countries developed national programmes for CHWs following the Alma-Ata Declaration on primary health care in 1978. CHWs are the first point of care and typically work at the household level [2]. They are widely employed across Africa and Asia and to a lesser extent South America and occasionally in the USA and UK. Typical roles include: nutrition, maternal and child health promotion, childhood immunization, infectious disease control and implementation of noncommunicable disease interventions [1].

The World Health Organization (WHO) supports the use of CHWs in countries such as Malawi with a shortage of health workers (two doctors per 100,000 people) and a high disease burden [3]. The WHO stresses the importance of having a national framework to guide task-shifting, clearly defined roles, consultation with all cadres, strong training, adequate supervision and regular assessment [4].

Several existing systematic [5,6] and nonsystematic reviews [7,8] provide evidence that CHW interventions can be effective in malaria prevention activities including the distribution of insecticide-treated nets and that CHWs can increase immunization coverage, improve breastfeeding rates, and show promising benefits in improving tuberculosis (TB) treatment outcomes and neonatal survival when compared to usual care. There is limited information regarding CHW training, supervision, prioritization of tasks, and challenges faced across multiple service delivery responsibilities. Amidst the research evidence, few studies have given voice to CHWs in low- and middle-income countries [9,10].

CHWs in Malawi are called Health Surveillance Assistants (HSAs). Initially they were called Cholera Assistants when first recruited in 1973 by the public health unit to fill a human resources gap and manage a large cholera outbreak [11]. Following the Alma-Ata declaration the position was renamed. In 1998, the Ministry of Health (MOH) recognized a growing need for the provision of health services at the community level and renamed Primary Health Care workers to the current title of HSA while making HSAs position within the health sector permanent [11,12]. HSAs are now employed within the Environmental Health Department [11]. According to the HSA job description, Assistant Environmental Health Officers are the formal supervisors of HSAs, however the more experienced Senior HSA position, also called HSA supervisor, provides the majority of direct supervision to HSAs [13].

According to the job description, the qualification of HSA is awarded to those who have completed the Malawi School Certificate of Education or Junior Certificate of Education and the MOH-approved HSAs’ pre-service training programme [13]. As the role of the HSA has evolved, so too has the HSA pre-service training programme. This was initially of six weeks duration but has been gradually increased to eight, then ten and now twelve weeks [11]. According to MOH documents, 5% of HSAs nationally are yet to receive pre-service training [11].

Today, HSAs comprise 30% of the health workforce in Malawi and they are often the only health workers serving rural communities where they are expected to reside [14]. The targeted ratio of HSAs to population is 1:1,000 [11]. The cadre is largely responsible for community-level delivery of the MOH Essential Health Package, being the minimum services provided to all Malawians free of charge [14]. Most recently, as part of the Essential Health Package, HSAs have been asked by the MOH to deliver community case management (CCM), an extension of the integrated management of childhood illness (IMCI) approach at the community level [15]. The MOH considers the three primary roles of the HSA to provide promotive, preventive and curative care; promote community participation in health care activities and to provide disease surveillance services at the community level [11].

It has been well documented that new activities such as microscopy and HIV testing and counselling are regularly being added both formally and informally to the HSA role and existing activities are being scaled-up using HSAs [13,16]. Generally, the effectiveness of scale-up programmes involving CHWs has been difficult to determine given the variation in the scale-up approach and the difficulties measuring outcomes [5-8,17]. However, locally the Médecins Sans Frontières experience in Thyolo district Malawi provides some evidence of feasibility and good outcomes for a programme of decentralized HIV care to clinics and community settings using task-shifting in which HSAs provided the HIV testing and counselling, and, treatment initiation was transferred to non-physician clinicians [18]. Between 2003 and 2009, the programme achieved universal access targets and achieved significant gains in human resources efficiency [18]. As such, the MOH continues to train and certify HSAs in HIV testing and counselling with the help of nongovernmental organizations (NGOs), such as Médecins Sans Frontières, who recognize the need for task- shifting this role to HSAs given the severe shortage of higher-level health workers [18]. The MOH has also reported that it aims to expand certain programmes, such as TB treatment, with HSAs as the primary providers [16].

A situational analysis of the HSA cadre in Zomba district Malawi was undertaken in order to comprehensively understand the HSA cadre’s role, training system and supervision. Reported here are the findings from the cross-sectional assessment of the HSAs’ performed versus documented roles and the examination of prioritization of tasks by HSAs, their supervisors and policy-makers.

## Methods

### Study design and setting

Zomba district in southern Malawi has over 670,000 inhabitants, 80% of whom live in rural areas [19]. Within Zomba district there are seven health service areas, known as clusters, containing 31 health centres. The study population included HSAs and HSA supervisors currently employed in Zomba district. Policy-makers within the MOH were also included to provide national-level perspective on the HSA system.

A qualitative design using ethnographic methods was used in order to gain a comprehensive understanding of the HSA cadres’ role, training, and supervision within Zomba district. Consistent with this methodology, data were collected via a review of documents, direct observation, field notes, HSA work diaries and semi-structured interviews and focus groups [20].

Peer-reviewed and grey literature related to the HSA cadre was reviewed. Documents were sourced from PubMed, Google and the Malawi MOH. Documents reviewed included previous and current HSA and HSA supervisor job descriptions, past and current HSA pre-service training curriculums and a review of the HSAs’ job description tabled to the Human Resources for Health Technical Working Group [13]. Interview guides were developed from the desk review and piloted with HSAs in the district to inform the final interview guides.

### Data collection

Data were collected in July and August 2012. A combination of random and purposive sampling was used to recruit participants. Further details regarding which groups were randomly selected and which were purposive are given below. In total, 75 of the 632 HSAs employed in Zomba district participated and were drawn from 17 of the 31 district health centres. Data were collected from fifty-five HSAs who participated in eight focus groups, fifteen individual HSA interviews, six policy-maker interviews, seven interviews with HSA supervisors, five HSA five-day work diaries and field notes from observations of three HSAs performing their duties over three days. Three HSAs who were observed or completed a work diary also participated in a focus group. Data collection continued beyond saturation until HSAs and their supervisors were recruited from all seven health service clusters.

For consistency of method, seven focus groups were drawn from each cluster’s largest health centre. An additional focus group was conducted in the central cluster at the main city clinic. The remaining HSA participants and HSA supervisors were selected from a further nine health centres chosen purposively by the research team with assistance from the MOH. At least one health centre from each cluster was chosen. Health centres were chosen based on size and distance from the main hospital. The MOH also used knowledge of monitoring reports to identify exemplar sites demonstrating strengths and exemplar sites demonstrating challenges in performance. Study sites were selected using these methods in order to capture heterogeneous social and geographical influences and gain an understanding of the breadth of challenges that HSAs encountered in fulfilling their role across the district.

**Figure 1:**
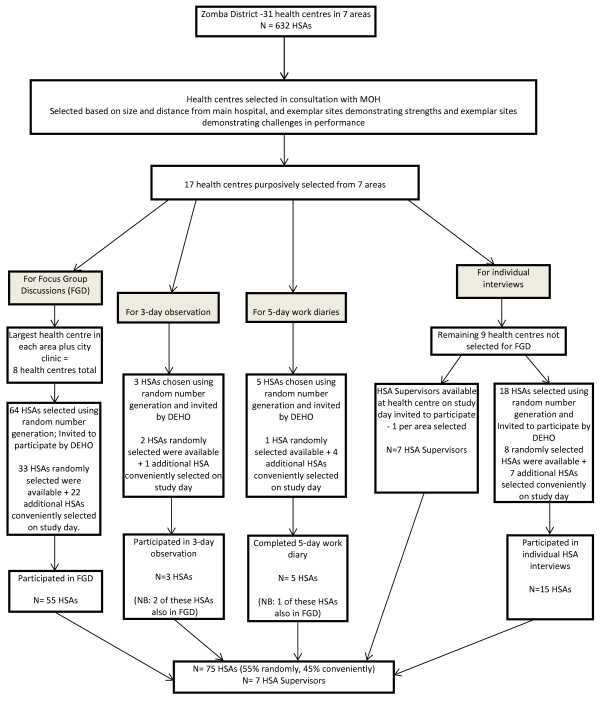
Selection of HSAs and HSA supervisors.

From chosen sites, a random number generator was used to randomly select HSAs to participate in the study from the total list of HSAs working at that site. HSAs were invited to participate by the District Environmental Health Officer (DEHO). When HSAs declined or were unavailable due to reasons such as leave, prior commitment or transfer, HSAs who were present and available to participate on the first day the researchers arrived at the health centre were recruited. HSA supervisors, known formally as Senior HSAs, were chosen from the centres selected for individual HSA interviews. HSA supervisors present on the study day were invited to participate until one supervisor from each area had been recruited. The selection of HSA and HSA supervisors is illustrated in Figure 1. Policy-makers were chosen purposively to include a range across HSA-associated MOH departments. For the purpose of the study, ‘policy-maker’ was defined as both a local and national-level decision-maker involved in the construction and operationalization of policy.

Interviews and focus groups with HSAs and their supervisors were conducted in the local language, Chichewa, by native Malawian research assistants fluent in both English and Chichewa. Policy-maker interviews were conducted in English. All interviews and focus groups used semi-structured interview guides, were audio recorded, transcribed *verbatim* and then translated into English. The five-day work diaries were completed in Chichewa and translated into English. Transcript translations were reviewed by a second research assistant to ensure accuracy. Malawian research assistants also conducted the participant observation and translated field notes into English.

### Analysis

A content analysis was employed whereby themes and sub-themes were derived directly from the data consistent with ethnographic methodology [21]. Investigators (SS, AD, NM, JB) read transcripts from interviews, work diaries and field notes independently, coding the data and clustering emerging themes and sub-themes. The team used a combination of NVivo 9 qualitative data software (QSR International Pty Ltd, Asia Pacific Head Office, 2nd Floor, 651 Doncaster Road, Doncaster, Victoria 3108, Australia) and manual data coding. Themes were discussed amongst the study team as they emerged to find consistencies and differences. Anomalies in the data were also identified so these could be further investigated in future interviews. In this way, investigators developed and synthesized the themes in an iterative process. Saturation was reached in that no new themes emerged from the data after three consecutive interviews. The major themes are presented in the findings. All quotes are by HSA respondents unless otherwise noted.

### Ethical considerations

This study was approved by the District Health Management Team in Zomba district and by the Malawi National Health Science Research Committee and the University of Toronto HIV Research Ethics Board. All HSAs, HSA supervisors and policy-makers participated voluntarily. Written informed consent was obtained from all participants in Chichewa or English. Quotes have been anonymized to protect the confidentiality of all participants.

## Findings

### HSAs’ performed versus documented role

The reported and observed experiences of HSAs, their supervisors and policy-makers was analysed and compared with the current job description and the HSA pre-service training curriculum to compare the HSAs’ performed versus documented role.

#### Task-shifting

Respondents acknowledged that the job description of HSAs had evolved and changed many times with the influx of new programmes and according to health priorities. Additional tasks were assigned informally to HSAs by both the MOH and local NGOs who coordinated the specific programmes. As such, the job description did not include the full range of tasks HSAs currently performed. One HSA stated:

‘Not all the works we do were written on the job description because there are other organizations which use us. So we cannot say that we only follow what was written to us by the government’.

In contrast, policy-makers indicated that the central MOH was responsible for task allocation across the health system. As one policy-maker noted:

‘Any activity before it is being done by the HSAs, the people on top there at the central level they sit down and decide who should this activity be given to; is it for clinical staff, is it for nursing, is it for environmental? So, once the people agree up there, then they just tell us and we have to tell them to do that job’.

Many HSAs saw the addition of tasks as inherent in the role as the job description stipulated ‘any other duty assigned’ such that they must comply when asked by seniors to perform a task outside their scope. One HSA said:

*‘*Sometimes some (tasks) we add which are not in the letter (job description) but when we are told to do them, we have to do’.

The additional tasks varied across Zomba district and between HSAs. The tasks not explicitly listed in the job description or taught comprehensively within the pre-service training curriculum being undertaken by HSAs in Zomba district were:

•Nutrition supplementation programmes for malnourished children at facilities;

•TB testing, drug dispensing, patient review;

•Dispensing and administering injectable contraceptives within family planning activities;

•Antiretroviral therapy for HIV - dispensing and monitoring, defaulter tracing and pre-antiretroviral therapy activities such as encouraging blood tests and engagement with health centres;

•Performing dry blood spot testing for infants within prevention of mother to child transmission of HIV (PMTCT) programmes

•Cholera management at health centres;

•Drug store management; and

•Outpatient register at the facility.

HSAs and policy-makers acknowledged that many of the additional tasks were activities traditionally and ideally performed by more qualified health workers. TB treatment activities, nutrition programmes, family planning and antiretroviral therapy activities were frequently identified and accepted by many HSAs as routine tasks within their role. One policy-maker stated:

‘In TB activities, they are the HSAs who are doing the work, they are the ones going out tracing the patients, they are the ones distributing drugs in the health facilities’.

The integrated management of childhood illnesses (IMCI) community case management (CCM) programme known as ‘village clinic’ was an example of a task recently added to the current HSA job description that was yet to be added to the pre-service training curriculum provided by the MOH. The programme required HSAs to assess children aged two months to five years in communities for common illnesses and provide basic treatments and referral:

‘Things pertaining to village clinics (IMCI CCM)…we know that they are supposed to be done by (higher-level) medical assistants but we do them. We know that it is their work, but bearing in mind that the government desires that people access assistance in their villages, we offer them. You may realize that in our villages we offer first treatment especially to the infants and this happens to be our additional task’.

Some HSAs expressed frustration that they were expected to take on tasks for which they were not qualified:

‘The work that I do but though am doing it I don’t feel happy with it is village clinic (IMCI CCM) because I am not a doctor and I was not trained for that (performing IMCI CCM) I am just a health worker (HSA)’.

HSAs did not only take on roles above their perceived skill set but also below. Some HSAs expressed frustration about performing duties such as cleaning at health centres or data collection outside of their normal role:

‘We went there and collect it without telling us what they want to do with the collected data. They just tell you a certain NGO wants it. Things like those are the ones I don't like’.

#### Preventive versus curative

Many HSAs spoke of a shift in their role from an environmental health and disease surveillance role to a role which included providing treatments. One policy-maker stated:

‘In the past they were more focused on preventive health, usually doing environmental health activities, as well as disease prevention and control, but now since they are also being involved in curative as well as family planning method and alike, it’s like now they are involved almost in all activities’.

For some HSAs the expansion of their role to include treatment tasks caused confusion:

*‘*Our main tasks is preventive so we are wondering that we are now doing the curative like the village clinics (IMCI CCM) - we have medicines’.

Opinion was divided amongst HSAs about the delivery of the IMCI CCM village clinics. Many saw it as a priority from a health promotion and prevention perspective as it reduced barriers to access, fitted well at the community level and communities valued it:

‘Another duty that satisfies me in the village is of village clinic (IMCI CCM) because parents get used to you when you are helping the kids. You being a fellow villager, they know you, get close to you; they trust you and they depend on you. Because they know that even if during the night a child falls sick, they rush to you knock on your door, and when you give them treatment’.

Others considered it clinical, not preventive, and therefore the responsibility of other cadres of health workers. Despite the majority of HSAs who participated in the study having received training for IMCI CCM village clinics via workshops, some felt insufficiently trained to perform the tasks and worried about the consequences to themselves and others by potentially misdiagnosing, mistreating or causing harm inadvertently:

‘We met different cases (diseases)…sometimes we fail to handle these just because of lack of knowledge or skills, and we also failed to help people even simple cases due to lack of knowledge’.

‘Giving medication in the village is a difficult work, it needs proper training in order to know what you are doing with your patients because medication sometimes are poison to patients, if you cannot give them relevant medication to the disease it can cause another disease’.

#### Community versus health centre-based

HSAs were being called upon increasingly to fill clinic-based human resources gaps and support health centre activities:

‘Due to task-shifting - where maybe some (higher-level) health workers are not available, that’s when you find HSAs providing some services at the facilities’.

Some saw an advantage in this as it enhanced links with centre-based services. Some HSAs felt confident and enjoyed doing centre-based tasks but others thought such tasks should not be allocated to HSAs:

‘They should not give us more programmes as HSAs just because they have a lot of pressures, for instance clinic work it’s not our part’.

The amount of time spent in clinics varied. HSAs in one centre spent one week per month away from their village catchment while in another, HSAs reported that 50% of their time was spent in facilities. A concern expressed by HSAs, their supervisors and policy-makers was that if too much time was spent filling gaps at the health centres, the preventive efforts in the catchment areas would suffer:

‘We get disturbed with clinic work because we have to be here for one week and another one week at (another centre), so two weeks at one month…sometimes we fail to work for the community’.

#### Training inadequate for tasks shifted

In addition to pre-service training, which HSAs had received to varying degrees, additional formalized training for new or specific tasks was being provided by the MOH and/or relevant NGO responsible for programme delivery. In view of their expanding role, most HSAs, supervisors and policy-makers felt that the training HSAs received was inadequate:

‘Relating to the work they are actually trained for…it seems it is adequate…But looking at the task-shifting, for the task-shifting issues they need to learn even more’. (Policy-maker)

Some of the additional tasks that had shifted to HSAs were performed without formalized training:

‘ART (antiretroviral therapy) we did it without proper training’.

‘I do work without any training…we deal with distribution of food to children who are malnutrition’.

HSAs reported that often not all HSAs received additional formalized training in new or specific tasks. Commonly, one or two HSAs at each centre were trained in new tasks such as administering family planning injections, and they then briefed their peers on how to perform them:

‘Like giving out TB medication, we just watch our friends who went for training even though we did not go for special training that this is how TB medication is given’.

‘Every Monday we must have a meeting…the one (HSA) who went to be trained on what has come have to brief the others (HSAs). That means when he has briefed us, we go and do what he has told us’.

There were varying opinions amongst HSAs on the adequacy of this seemingly informal peer-led training. Some HSAs commented that they were performing tasks confidently after being trained by a peer on-the-job. A few HSAs and a policy-maker expressed a fear that informal on-the-job training could result in HSAs passing on incorrect information or techniques. One HSA suggested training more than one HSA at each site to enhance the quality of on-the-job peer-led training:

‘They send information through someone we did not get it clear, so they found weakness that we don’t do our work. May be the reason can be a person did not get information clear…they should train two or three, five people it can be good because it can help us to get the information clearly’.

Some HSAs expressed frustration with what they saw as a misallocation of training to other cadres, particularly nurses who received longer training and larger allowances while HSAs implemented the activities at the grass roots level:

‘So we wanted that training because for that training, they mostly just focus on nurses but when it is for work, it comes to us the village workers (HSAs)’.

Many HSAs said they were not competent or happy to perform tasks for which they had not been trained:

*‘*Every job that we have been taught, it is the one that we happily carry out. As compared to the job may be they have given us that you should do this job, we do not know it and we have never learnt, it is the one that we are not happy with.*’*

#### Task-shifting without appropriate supervision structures

HSAs, HSA supervisors and policy-makers all identified weaknesses in the supervision of HSAs and this was seen as a significant issue particularly when new tasks were being added to HSAs’ duties. Policy-makers in particular highlighted a lack of integrated supervision, even across MOH departments as a constraint to HSA effectiveness:

‘I don’t know what other supervisors are doing…we don’t go as an integrated programme. …it’s usually maybe the nursing (department)…supervise them on the clinical component…maybe the preventive (department) will go also’. (Policy-maker)

‘So you will find that the HSA has got multiple supervisors…There is the Assistant Environmental Health Officer or Environmental Health Officer who is supposed to be the supervisor....But depending on what (tasks) they have given him…the HSA is not supervised proper because each programme manager, each programme coordinator, wants to supervise an HSA on the work that his programme has given the HSA’. (Policy-maker)

One policy-maker said that the Assistant Environmental Health Officers and Environmental Health Officers who supervised the HSA supervisors were not receiving training for tasks shifted to HSAs, thus restricting their capacity to effectively supervise HSAs and HSA supervisors:

‘We are targeting the HSAs…these other cadres of their supervisor the Environmental Health Officer, the Assistant Environmental Health Officer; they are being left out…How can you supervise something you don’t know?’

#### Lack of remuneration or resources for shifted tasks

Participants noted that while the role had expanded, the remuneration had not increased accordingly and HSAs who performed specialized tasks were not receiving allowances:

‘Another thing that I see as a problem on our work is the abundance of the work which we receive to do, comparing to what they employed us for. So it seems we do a lot of work but we receive peanuts’.

‘That health worker (HSA) is doing probably the same that the nurse could do but the nurse is getting more so we go into that type of conflict’. (Policy- maker)

Most HSAs in this study felt that the remuneration they received did not reflect the value of their role and considered it a sign that the MOH did not value their contribution. Many HSAs said their role was hampered by a lack of medical supplies, office supplies, protective equipment, bicycles and mobile phone credit.

#### Role confusion

Contributing to confusion around the scope of the HSA role was that many - 30 of 70 HSAs asked - did not have a copy of their current job description. They referred to their training notes and curriculum as outlining their role or had seen previous versions only. Neither accurately reflected the current MOH job description for HSAs:

‘We don’t have a letter of our job description they only brief us what we have to do’.

### Prioritization of HSA tasks

#### Priority on prevention and community

Consistent with the job description, HSAs frequently described their job as ‘community- based’ and ‘grass roots’ within a designated catchment area. They spoke about their role in building relationships with village leaders and the communities they serviced, as well as encouraging community participation in health care. Many HSAs and policy-makers referred to HSAs as a bridge or link between communities and the health system.

HSAs, HSA supervisors and policy-makers agreed that the HSAs’ preventive role in the community was most important and should remain a future priority. They recognized the potential to reduce disease burden, mortality, morbidity and demands on overburdened health facilities. An economic benefit to prioritizing prevention was recognized also:

‘My plea is that we should continue giving them (HSAs) the right support and doing more on preventive health services. You know prevention is cheaper and is simple…’(Policy-maker)

Many HSAs expressed pride in the importance of their prevention role and the benefit of their work to the population:

‘When we hear that this year in our country the report reveals that there is zero outbreak of such diseases we become very happy…when there is zero report it means that the one who has tried to do a very important job is we the HSAs in the community’.

#### Priority on access and equity

Because of their location in the communities, their numbers and skill mix, there was a perception that HSAs had the potential to effectively and efficiently provide more equitable access to services:

‘Those people (HSAs) are very close to the community so for any public health intervention they are close to the people and they would carry that very well and dissemination of that promotion would be even faster and more effective’. (Policy-maker)

HSAs valued their role in improving access to health care and they prioritized community health needs:

‘I organized a meeting with the villagers last week, they thanked me that this time around more children are not walking long distances because at first they were going to the (health centre) but now they are accessing the services in their village and this has to continue’.

‘People were not attending (health centre) because they were not having money for the treatment so today we are happy because we are helping them freely not paying so cases like people delaying to go to hospital may be children dying at a household are few…because we are giving them medications’.

Many HSAs wanted training to provide additional essential services such as HIV testing and counselling in their communities where they identified a need and demand:

‘If possible government should teach all HSAs in HTC (HIV testing and counselling) and family planning in order to help people…what happens when we are working in the community giving treatment, people ask us if we can provide to them these services, so like to my village, the health centre is very far and other people just stay without knowing their (HIV) status’.

### Task-shifting and prioritization challenges

#### Overloading

Many HSAs felt they had been assigned too many tasks by the MOH and local NGOs such that they were overloaded:

‘Since we are in the village, right, they depend on us. They know that it is us who live with the children from the time they are very young. So, whatever programme comes, comes to whom? To us’.

Some HSAs said they were unable to fulfil their job description and neglected to perform some activities because of the additional tasks.

‘These other activities consume much of our time and instead our normal duties do not bear results in good time as we are preoccupied with other duties’.

Some HSAs felt they had too many jobs to be able to do them all well:

‘We have a lot of jobs, so for you to become an expert at a particular job it becomes difficult because if you are to do a job you should put your heart into it, another one comes that you should do such, so we have a lot of jobs so that it becomes difficult to pick out one job that we are good that, the jobs are just too many’.

In contrast, some policy-makers believed HSAs were not overloaded. They believed poorly organized and non-integrated programmes and ineffective supervision made it difficult for HSAs to incorporate additional tasks and manage their workload:

‘They (HSAs) just need to be mentored to make proper programmes for the community interventions…they just have to organize themselves and make a good programme for that…they will be complaining; no, we are loaded with a lot of roles. But if you could critically review what they have done in a month…you just see that it just need organization’. (Policy-maker)

Some policy-makers believed that many additional tasks were easily incorporated and offered when conducting routine community activities:

‘You are in this community you are teaching the people about family planning, why don’t you spare some minutes to also teach these people about sanitations?’ (Policy-maker)

#### Specialization

Specialization occurred when some HSAs received additional training which allowed them to perform specific ‘specialized’ activities such as HIV testing and counselling, drug store management, and TB sputum microscopy testing:

‘Sometimes it overloads. For example in my case, I did training in drug store management; it needs you to be there full time. If you have been assigned three days there, you have to make sure that everything there is working. It at the same time happens that in the field the work is intense as well, things are not working’.

‘So, when time comes to conduct our normal duties, you find that the same time you are required to attend clients at the office as a counsellor. It happens that my friends do worry because I am unable to perform my duties as HSA, it affects us a lot’.

Some policy-makers believed specialization of HSAs was required to meet health priorities:

‘Why not specialize if we want some HSAs to be doing family planning, they should look into that’.

Other policy-makers feared that specializing HSAs risked losing the benefits of a broad-based community cadre focused on prevention and health surveillance:

‘My fear is if they become specialized, we risk to them…they were supposed to keep a watchful eye on what is happening in the community setting’.

Specialization caused differences within the cadre which may have implications for remuneration:

‘Are we giving this HSA new title? Or are we keeping him as an HSA? Now how different is this HSA to the rest of the HSAs?’ (Policy-maker)

Many HSAs believed that all HSAs should be trained in current specialized activities as opposed to specialization. In this way, HSAs saw that they could support colleagues in sharing duties at the facilities:

‘So, it could be better if more counsellors were trained so that any time the HSA is here (health centre), he or she would have knowledge to do the work’.

One policy-maker highlighted the disparity and possible tensions amongst HSAs that may occur with increased specialization and suggested an alternative to recruiting and specializing more HSAs was needed:

‘We need to look at the issue of task-shifting and we aren't asking are we shifting the tasks to only a few group of HSAs and then they form their own colony and consider themselves different from the other HSAs that are working in the general community setting’.

HSAs highlighted a potential negative consequence of specializing:

‘We do face some challenges that when they see a health worker HSA they think that he or she was trained in everything…it could be better if they train all of us on family planning services so that our community could access the services without difficulties’.

#### Competing priorities at the local level

HSAs spoke of multiple programmes and supervisors at the local level placing competing demands on them to prioritize their activities. Policy-makers noted the lack of integration of NGO programmes and the implications this had on task-shifting and prioritization locally:

‘You find that some of the NGO they don’t do integration, they just want their programme to be done at that particular time…managers are biased towards their programme because everyone wants his programme to work. Yeah, so with that thing, it is like the HSAs now start losing focus to other activities which also they are supposed to implement’. (Policy-maker)

Policy-makers said tasks were being prioritized almost exclusively over others depending on demands made or incentives offered from well-funded programmes, thus diverting HSAs from core community duties. In this way, specialization of the HSA may have been an unintended consequence of disease-specific programming:

‘That’s why the HSA sometimes, may focus on only one area probably because that area is well funded’. (Policy-maker)

Several policy-makers expressed concern that the MOH did not have ‘full control’ of tasks allocated to HSAs. One respondent said NGOs had been bypassing the district supervisor of HSAs and directly recruiting HSAs for their programmes. As such, a directive was issued in July 2012 advising local organizations that access to HSAs must be arranged via the District Health Management Team. One policy-maker raised concerns about the sustainability of local NGOs using HSAs to implement programme-specific activities:

‘One of the (programmes) was HIV/AIDS, it was well funded, well supported. Those they were coordinating the programmes were attracting HSAs often, they needed data, needed this and that…I think the project was very attractive to the health workers…until issues of sustainability came up and they started scaling down’.

## Discussion

The HSA role in Malawi has been evolving as the health needs and the capacity of the health system to provide services has changed. Being the only community-based health care cadre, a link between villages and health centres, and a sizeable workforce has meant HSAs have the potential to provide more equitable access to health services. HSAs have enabled the MOH to push forward specific health agendas, particularly the Essential Health Package, which placed them at the forefront of large scale prevention campaigns such as childhood immunization through the Enhanced Programme of Immunizations and malaria campaigns. Most HSAs, HSA supervisors and policy-makers felt preventive tasks were and should remain the priority for HSAs. They appreciated the potential benefits in reducing the burden of disease and mortality and recognized the economic advantages for a strained health system.

However, the role of the HSA has continued to expand beyond prevention to include tasks that are curative in order to respond to disease priorities. The MOH has now listed ‘curative’ in the definition of HSAs’ primary role [11], however some HSAs remained adamant that it was not within the scope of their practice to offer treatments. There were varying opinions about whether HSAs should perform curative tasks amongst both HSAs and policy-makers.

Due to shortages in human resources, there has been an increasing demand on HSAs to perform health centre-based duties such as microscopy, drug management, under fives clinics and HIV testing and counselling. While having an interface between community and health centres was considered essential to effectively fulfilling their function, this study highlighted HSA and policy-maker concerns that increased time spent in health centres results in neglect of community-based prevention tasks. The Malawi-appointed task force review of HSA job descriptions in 2011 concluded that HSAs spent on average 65% of the time in the communities and 35% in the health centres [13]. The MOH argued that this was not the intended role of the HSA and ‘something needs to be done in order not to lose the direction of having the HSAs as community-based health workers’ a sentiment echoed by both HSAs and policy-makers in this study [11], p.12.

HSAs were being used across Zomba district by NGO and MOH programmes to provide a range of services beyond the scope of the current job description. The task force review of the HSAs’ job description suggested including HIV and TB defaulter tracing and treatment monitoring activities, HIV testing and counselling, pre-antiretroviral therapy activities, dry blood spot sampling tests for malaria and family planning interventions in a revised job description given HSAs were perceived to be performing the tasks routinely and safely across Malawi [13]. Most policy-makers noted that IMCI CCM village clinic and many of the additional tasks HSAs performed, would ideally be provided by more highly qualified personnel but HSAs were considered the most appropriate cadre currently and could not be removed from the essential tasks until there was a significant increase in numbers amongst other cadres at health facilities [13].

Including additional tasks formally in a revised job description and ensuring all HSAs are familiar with their job description would likely serve to reduce confusion and resultant frustration for HSAs which was evident in this study. A revised job description would clarify what is and is not the role of the HSA; a condition necessary for improved worker satisfaction and morale [22]. Interestingly, this study gives a sense of the willingness of HSAs to expand and even change the scope of their practice in response to heath priorities given appropriate training and resources. Previous global work has shown that meeting community expectations, engaging with communities and providing communities with services they consider valuable remains essential to the success of any ongoing CHW programme [8].

Most policy-makers and many HSAs felt they were being given more tasks than they could handle which distracted them from their intended roles in the community. Several other studies have also found that the ever-increasing workload was a serious issue for HSAs [11-13,23]. Attention needs to be paid to this issue, as effective task-shifting is unlikely if HSAs are overwhelmed by the number of tasks [22]. Continued focus is needed in ensuring HSAs have access to vital resources such as transport, mobile phones and accommodation that would increase efficiency and capacity to perform the larger role [22].

This study raised questions about whether to continue training HSAs broadly and then have some focus on certain task such as HIV testing and counselling or whether to recruit and train workers specifically for HIV programmes for example, so HSAs could remain broad-based community, preventive health workers. It is recognized in the literature that a lengthy and unresolved debate has continued in regards to specialization of CHWs in terms of how many tasks one can carry out given the scope of potential activities along with questions about what the primary role actually is [1,8]. It has been suggested that specialized CHW roles may occur due to the difficulty governments have in finding the optimum mix of roles and tasks and the ideal balance between depth and breadth of tasks [8]. This current dilemma for policy-makers on the specialization of HSAs warrants further research and debate - especially in light of the new moves to also have HSAs take on roles in chronic disease surveillance and management [24,25]. It is worth noting that none of the written, observed or collected data in this study referred to chronic diseases despite these increasingly accounting for much of the disease burden in low- and middle-income countries [26].

Although NGO tasks were seen as complementing government efforts, the lack of integration, vertical programming and multiple supervisors associated with NGO programmes meant competing demands on HSAs to prioritize activities which contributed to overloading and compounded role confusion. In this study, there was evidence that pressures existed at the community level to prioritize disease-specific programmes over the core duties outlined in their job descriptions. There is concern in the literature about the negative consequences to the broader health system of global health initiatives which have a disease- specific focus, in particular those related to the scale-up of HIV activities [27]. It is also recognized that specialization is often an unintended consequence of such disease-specific programming [8]. Some HSAs struggled to meet the demands of their role because of distraction from, or even diversion entirely to, programme-specific activities.

Policy-makers expressed concern about the lack of policy and MOH ‘control’ that had resulted in the fragmentation of programmes, training and supervision. There is evidence in the literature that effective task-shifting requires leadership from governments in establishing policies, engaging stakeholders, supporting training, and providing adequate resources [28]. There was a feeling amongst policy-makers which is echoed in the literature, that better organized programmes and improved supervision would allow HSAs to manage the work load more effectively and potentially see the benefits of the HSA cadres’ role applied to a greater range of health priorities [8,22]. Improved collaboration within the MOH itself and improved collaboration between the MOH, other government departments and NGOs in planning and implementing programmes may improve integration and the capacity of HSAs to meet the demands of an ever expanding role. The IMCI CCM village clinic could serve as a model for other programmes as it has demonstrated effective collaboration between sectors and government departments, is strongly led by the MOH, and its policy directs the work of partners and provides a framework for the coordination and partnership of many organizations [15].

This study’s findings have been actively disseminated to various HSA stakeholders within Malawi, including the Zomba District Health Management Team and national-level HSA policy-makers from the Essential Health Package Technical Working Group - a key HSA policy-making forum.

## Conclusion

This study provides insights into the expanding role and the nature of task-shifting and job task prioritization in relation to HSAs’ duties in Zomba district, Malawi. The role of the HSA has continued to expand in an *ad hoc* manner across the district and health centres. This study provides further evidence that task-shifting often occurs without policy support and takes place in an environment where there are competing demands, inadequate training and resources, and inadequate supervision structures as a result of non-integrated, disease-specific programming. For HSAs, confusion regarding their role creates frustrations and cadre wide inefficiencies. The study shares HSAs’ perceptions of their work, their experiences of task-shifting, and gives a sense of their willingness to change the scope of their practice in response to health priorities. For policy-makers there are a range of prioritization and task allocation considerations in regards to the HSAs’ role in the health system including treatment versus prevention, facility versus community, specialization versus broad-based roles and infectious diseases versus chronic disease management.

## Abbreviations

CHW: community health worker; CCM: community case management; DEHO: District Environmental Health Officer; HSA: Health Surveillance Assistant; IMCI: integrated management of childhood illnesses; MOH: Ministry of Health (Malawi); NGO: nongovernmental organization; TB: tuberculosis; WHO: World Health Organization.

## Competing interests

The authors declare that they have no competing interests.

## Authors’ contributions

Conception and design: AM, JB; Analysis and interpretation of the data: SS, AD, JB, NM; Drafting of the article: SS, AD, JB, JN, NM; EC; Critical revision of the article for important intellectual content: AM, JN, LMPR. All authors read and approved the final manuscript.

## References

[B1] Global Health Workforce Alliance and WHO Global Experience of Community Health Workers for Delivery of Health Related Millennium Development Goals: A Systematic Review, Country Case Studies, and Recommendations for Integration into National Health Systems 2010 Geneva: World Health Organization

[B2] The Earth Institute One Million Community Health Workers: Technical Task Force Report 2011 Columbia: Columbia University

[B3] WHO Country Health Profile: Malawi 2013 Geneva: World Health Organisation

[B4] WHO Task Shifting: Rational Redistribution of Tasks Among Health Workforce Teams: Global Recommendations and Guidelines 2008 Geneva: World Health Organisation

[B5] Christopher JB Le May A Lewin S Ross DA Thirty years after Alma-Ata: a systematic review of the impact of community health workers delivering curative interventions against malaria, pneumonia and diarrhoea on child mortality and morbidity in sub-Saharan Africa Hum Resour Health 2011 9 1 27 10.1186/1478-4491-9-27 22024435 PMC3214180

[B6] Lewin S Munabi-Babigumira S Glenton C Daniels K Bosch-Capblanch X van Wyk BE Odgaard-Jensen J Johansen M Aja GN Zwarenstein M Scheel IB Lay health workers in primary and community health care for maternal and child health and the management of infectious diseases Cochrane Database Syst Rev 2010 Issue 3 CD004015 doi:10.1002/14651858.CD004015.pub3 20238326 10.1002/14651858.CD004015.pub3 PMC6485809

[B7] Haines A Sanders D Lehmann U Rowe AK Lawn JE Jan S Walker DG Bhutta Z Achieving child survival goals: potential contribution of community health workers Lancet 2007 369 9579 2121 2131 10.1016/S0140-6736(07)60325-0 17586307

[B8] Lehmann U Sanders D Community Health Workers: What do we Know About Them? The State of the Evidence on Programmes, Activities, Costs and Impact on Health Outcomes of Using Community Health Workers. Evidence and Information for Policy 2007 Geneva: World Health Organization

[B9] Daniels K Van Zyl HH Clarke M Dick J Johansson E Ear to the ground: listening to farm dwellers talk about the experience of becoming lay health workers Health Policy 2005 73 1 92 103 10.1016/j.healthpol.2004.10.006 15911060

[B10] Mock J Nguyen KH Bui-Tong N McPhee SJ Processes and capacity-building benefits of lay health worker outreach focused on preventing cervical cancer among Vietnamese Health Promot Pract 2006 7 3 Suppl 223S 232S pub 7 June 2006 10.1177/1524839906288695 16760246

[B11] MOH The Health Surveillance Assistants: Origins and Current Status 2012 Lilongwe, Malawi: Ministry of Health

[B12] Katsulukuta A Improve Coverage: Opportunities and Challenges 2010 Geneva: The Global Immunization Meeting

[B13] Nkhono E Banda A Kalilangwe J Report on the Taskforce on the Review of Job Descriptions of HSAs 2011 Lilongwe, Malawi: Ministry of Health

[B14] MOH Human Resources for Health: Malawi Country Profile. Malawi Health Workforce Observatory 2010 Lilongwe, Malawi: Ministry of Health

[B15] Fullerton JT Schneider RM Aurukun A Malawi Community Case Management Evaluation 2011 Washington: USAID

[B16] Carlson C Boivin M Chirwa A Chirwa S Chitalu F Hoare G Huelsmann M Ilunga W Maleta K Marsden A Martineau T Minett C Mlambala A von Massow F Njie H Olsen IT Malawi Health SWAp Mid-term Review 2008 Lilongwe, Malawi: Ministry of Health

[B17] Pallas SW Minhas D Pérez-Escamilla R Taylor L Curry L Bradley EH Community health workers in low- and middle-income countries: what do we know about scaling up and sustainability? Am J Public Health 2013 103 7 e74 10.2105/AJPH.2012.301102 23678926 PMC3682607

[B18] Bemelmans M Van den Akker T Ford N Philips M Zachariah R Harries A Schouten E Mwagomba B Massaquoi M Providing universal access to antiretroviral therapy in Thyolo, Malawi through task shifting and decentralization of HIV&frasl;AIDS care Trop Med Int Health 2010 15 12 1413 1420 10.1111/j.1365-3156.2010.02649.x 20958897

[B19] National Statistical Office Population and Housing Census Report 2008 Malawi: National Statistical Office

[B20] Creswell JW Qualitative Inquiry and Research Design: Choosing Among Five Approaches 2007 2 Thousand Oaks: Sage Publications

[B21] Spencer L Ritchie J O’Connor W Ritchie J, Lewis J Analysis: practices, principles and processes Qualitative Research Practice: A Guide for Social Science Students and Researchers 2005 London: Sage Publications 199 218

[B22] Jaskiewicz W Tulenko K Increasing community health worker productivity and effectiveness: a review of the influence of the work environment Hum Resour Health 2012 10 38 10.1186/1478-4491-10-38 PMC3472248 23017131

[B23] Kadzandira J Questioning the Capability of Health Surveillance Assistants to Deliver Activities of the Malawi Essential Health Package. Inter-Stat Report Number 25 2002 East Kilbride: Department for International Development

[B24] El-Sadr WM Abrams EJ Scale-up of HIV care and treatment: can it transform healthcare services in resource-limited settings? AIDS 2007 21 Suppl 5 65 70 10.1097/01.aids.0000298105.79484.62 18090271

[B25] Rabkin M El-Sadr WM Why reinvent the wheel? Leveraging the lessons of HIV scale-up to confront non-communicable diseases Global Public Health 2011 6 3 247 256 10.1080/17441692.2011.552068 21390970

[B26] Samb B Patel K Adshead F McKee M Evans T Alwan A Etienne C Desai N Nishtar S Mendis S Bekedam H Wright A Hsu J Martiniuk A Celletti F Prevention and management of chronic disease: a litmus test for health-systems strengthening in low-income and middle-income countries Lancet 2010 376 9754 1785 1797 10.1016/S0140-6736(10)61353-0 21074253

[B27] Biesma RG Brugha R Harmer A Walsh A Spicer N Walt G The effects of global health initiatives on country health systems: a review of the evidence from HIV/AIDS control Health Policy Plan 2009 24 239 252 10.1093/heapol/czp025 19491291 PMC2699244

[B28] Lehmann U Van Damme W Barten F Sanders D Task shifting: the answer to the human resources crisis in Africa? Hum Resour Health 2009 7 49 10.1186/1478-4491-7-49 PMC2705665 19545398

